# Supplementary figures and images for: Examining the viability of five Salmonella enterica subsp. enterica in thymol at 4°C and 25°C using flow cytometry
Source: PLoS One. 2025 Sep 25;20(9):e0332053. doi: 10.1371/journal.pone.0332053 (PMC12463282; doi:10.1371/journal.pone.0332053)

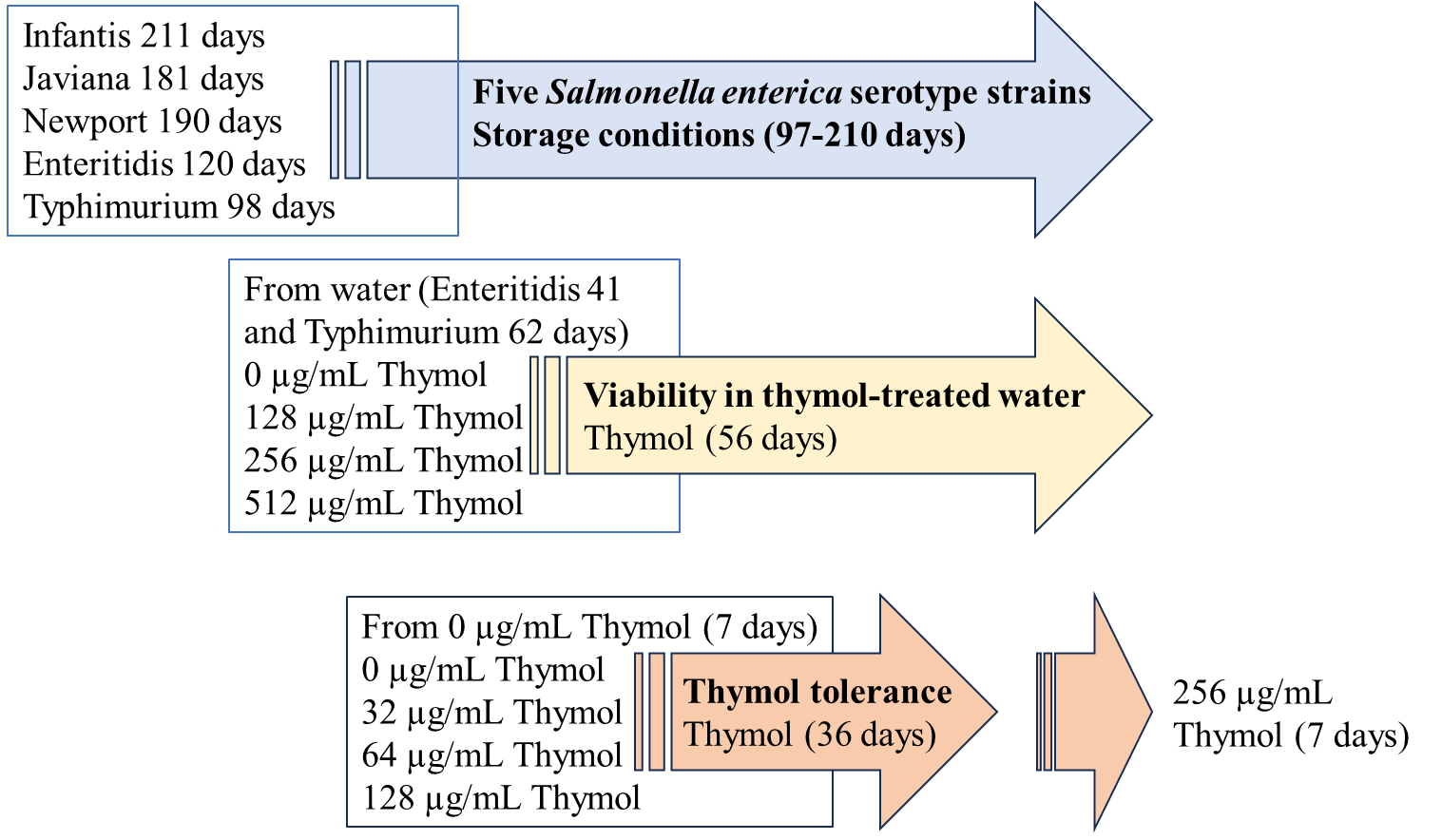

Supplement: S1 Fig — This figure visually represents the sequence of experimental steps involving Salmonella enterica serotype strains subjected to different treatments over varying durations. The flow chart consists of arrows, each corresponding to a specific phase of the experiment. Phase 1: Initial storage and incubation. Phase 2: Viability in thymol-treated water. Phase 3: Thymol tolerance testing. (DOCX) [file pone.0332053.s001.docx]

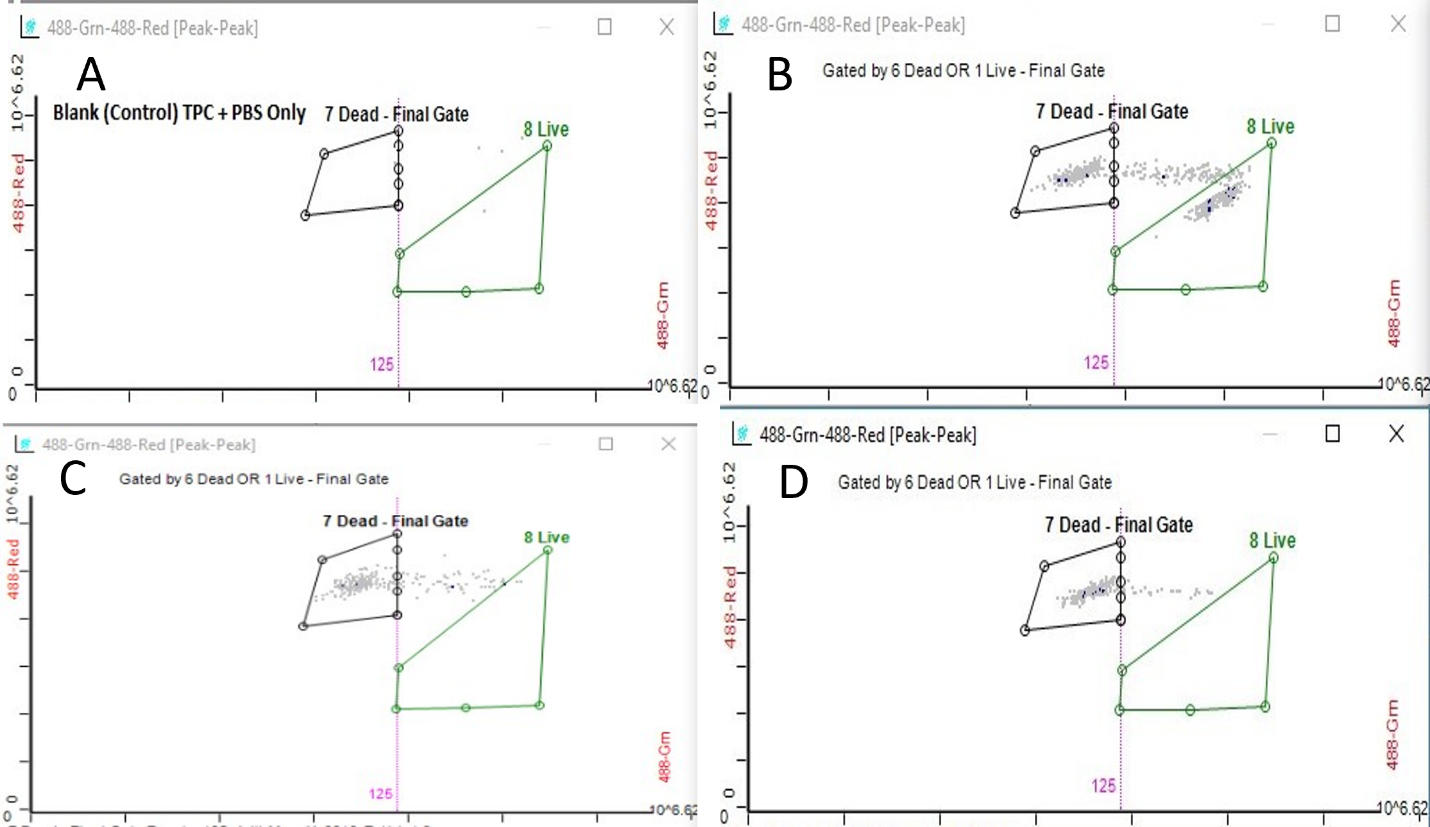

Supplement: S2 Fig — (A) blank sample containing phosphate buffer (PBS) and total plate count (TPC) reagent alone; (B) Day 7 – S. Enteritidis with sub-MIC 128 μg/mL thymol; (C) Day 7 – S. Enteritidis with sub-MIC 256 μg/mL thymol; and (D) Day 7 – S. Enteritidis with sub-MIC 512 μg/mL thymol. (DOCX) [file pone.0332053.s002.docx]

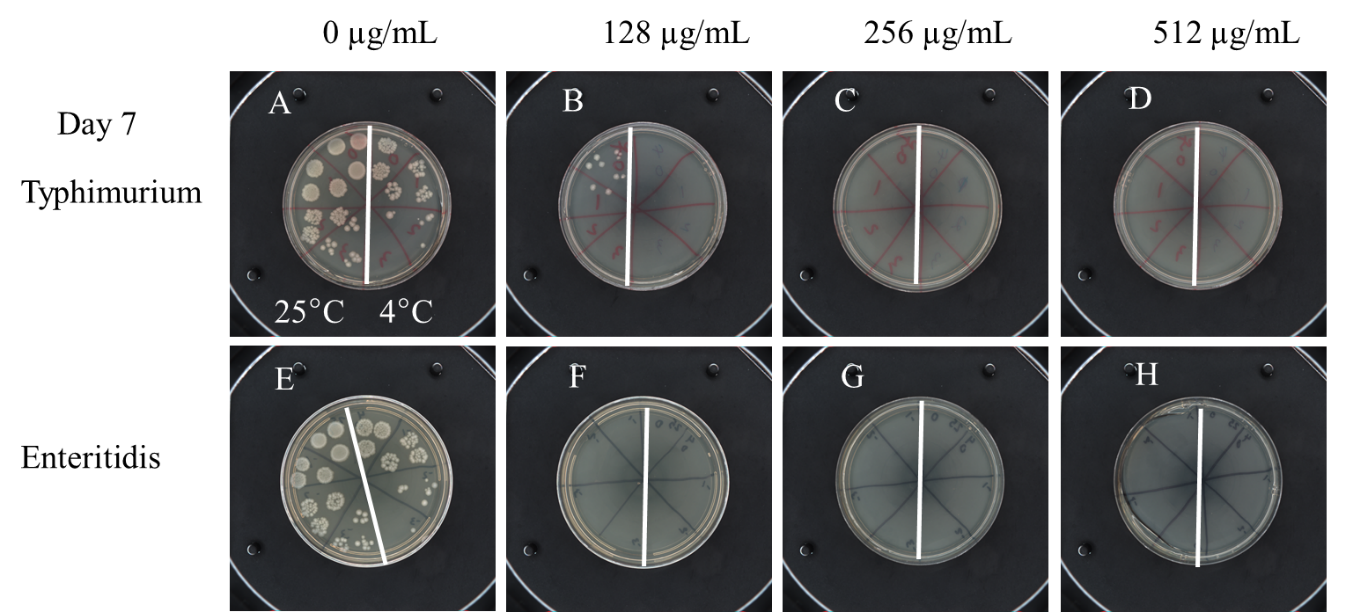

Supplement: S3 Fig — Salmonella Typhimurium (A, B, C, and D) and Enteritidis (E, F, G, and H) exposed to 0 µg/mL (2% ethanol, A and E), 128 µg/mL (B and F), 256 µg/mL (C and G), and 512 µg/mL (D and H) of thymol after 7 days on TSA plates at 25oC and 4oC. The left side of each plate was inoculated with cells incubated at 25oC and the right side of each plate inoculated with cells incubated at 4oC. For comparison with various concentrations of thymol, a series of four dilutions was plated (three times) at 25oC and 4oC and imaged using the ProtoCOL3 automated plate counter. (DOCX) [file pone.0332053.s003.docx]
