# Supplementary material for: Examining the viability of five Salmonella enterica subsp. enterica in thymol at 4°C and 25°C using flow cytometry
Source: PLoS One. 2025 Sep 25;20(9):e0332053. doi: 10.1371/journal.pone.0332053 (PMC12463282; doi:10.1371/journal.pone.0332053)
Supplement: Attachment — Submitted filename: Response to reviewers.docx [file pone.0332053.s004.docx]

**Reviewer #1:**

Abstract -if have word count available- give an example of types of products thymol is used in if any
We appreciate this valuable comment! Unfortunately, we have not word count available in Abstract. We have added the following statement in the Introduction section on **lines 66-68**, namely. “Thymol is also included in various personal care products for its antimicrobial and aromatic qualities. This includes hand sanitizers, soaps, acne treatments, and some topical creams and ointments for skin infections.”

Introduction-
-Italicize salmonella names-check this overall in text

As recommended, we have italicized Salmonella names. However, serotypes should not be put in italics.

-Line 77 include Salmonella in all serotypes line just so clear you mean all salmonella serotypes also our lab sounds more professional than we if talking about previous studies not just current one.

Thank you for pointing this out! Since we have provided references, we though it to be superfluous to specify which *Salmonella* serovars we were referring to (see Line 81). We have therefore used “our lab” instead of “we” to maintain a professional tone.

-Is thymol just used in water? Is there more products it is in? Does this currently have a commercial use or is it just studied alternative. This is not clear.

We appreciate this valuable comment! We have added the following statement in the Introduction section on **lines 66-68**, namely. “Thymol is also included in various personal care products for its antimicrobial and aromatic qualities. This includes hand sanitizers, soaps, acne treatments, and some topical creams and ointments for skin infections.”

Materials and Methods:
-no comments
Results
-no comments
Discussion + conclusions
-no comments

Figures
-figures need titles and figure legends. Hard to understand currently

We added separate captions for each figure in the manuscript.

**Reviewer #2:** This study provides valuable insights into the antimicrobial effects of thymol on Salmonella enterica, with potential applications in food and water safety. However, the following revisions are required to strengthen the manuscript:

1. Statistical analysis: The methods section should specify the number of replicates used in the experiments. Statistical significance (e.g., p-values) must be clearly reported to support the conclusions. Provide error bars in the graphs.

As recommended, we have added the following statement in the Material and Methods section on **lines 195-199**, “**Statistical analysis** Graphs represent averages of triplicate samples. Significant differences between the detection of intracellular materials (nucleic acids and protein materials), number of colony forming units and TPC assay using RAPID-B flow cytometry for *S.* Typhimurium and *S.* Enteritidis were determined using SigmaPlot vs. 14.1 software (GraphPad Software, Inc. USA).” Furthermore, we added in the Fig. 1 legend the following description: “The graphs indicators were significantly lower in the 0 (2% ethanol), and 32 µg/mL than in the 256 µg/mL based on the result of the Mann-Whitney Rank Sum test (*p* < 0.05).” and Fig. 4 legend the following description: “The graphs indicators were significantly lower in the 0 (2% ethanol), than in the 128 (sub-MIC) µg/mL (*p* < 0.05).

2. Data interpretation: The observed reduction in protein materials and increase in nucleic acid content requires further explanation. Please discuss the possible biological mechanisms underlying these changes.
As recommended, we have added the following statement in the Discussion section on **lines 334-341**, “Thymol disrupts bacterial cell membranes primarily due to its lipophilic nature. Its structure allows it to insert itself into the lipid bilayer of the bacterial membrane. Thymol's presence at the bacterial cell membrane disturbs the tightly packed arrangement of lipids, which increases the membrane's fluidity and permeability [35]. This instability leads to the leakage of essential intracellular materials (*i.e.*, nucleoid, ribosomes, plasmids, RNA molecules and larger proteins) as shown in Fig. 2C. At higher thymol concentrations, the membrane disruption becomes so severe that it causes visible structural damage to the cell surface [36], as seen in SEM micrographs. This can lead to the observed cell lysis, or bursting.” We believe this information sufficiently addresses the concern raised by the reviewer.

3. Practical implications: The Conclusion section should explicitly address how these findings could be applied in real-world food safety or disinfection protocols.
For clarification, we have improved the following sentence in the Conclusion on **lines 383-392**. “Therefore, this study is in favor of using advanced techniques such as flow cytometry and highlights thymol's potential as a safe and inexpensive antimicrobial agent. In food safety, thymol's Generally Recognized as Safe (GRAS) status by the FDA allows for its use as a preservative in certain foods. Its efficacy and low toxicity is conducive to its use as a preservative and disinfectant. As a preservative, thymol can be incorporated into food products or packaging to inhibit the growth of common foodborne pathogens such as *Salmonella* spp., thus extending shelf life and enhancing food quality assurance. For work surfaces, thymol is a key active ingredient in botanical disinfectants and sanitizers, providing an alternative to traditional chemical agents. Thymol-based products are effective for decontaminating food-contact and non-food-contact surfaces, reducing the risk of cross-contamination in food processing facilities, kitchens, and other environments.”

4. Please highlight the advantages of flow cytometry over viable plate count method.

Based on the text that appears on **lines 348-352**, namely “The RAPID-B TPC flow cytometric assay can distinguish between live and dead cells [20, 25]. Most recently, the RAPID-B TPC flow cytometric assay, when compared to traditional growth plates, demonstrated that *Salmonella* serotypes are capable of surviving in distilled water for over 160 days at both 25°C and 4°C [20]. In this study, *Salmonella* serotypes treated with thymol failed to grow on TSA plates, but were detected using the RAPID-B TPC assay.” We believe this information sufficiently addresses the concern raised by the reviewer.

5. Language editing: There are some grammar errors (for instance line 180). A thorough grammar and style review is recommended to improve readability (e.g., subject-verb agreement, article usage).

Thank you for your suggestion! To ensure clarity and precision, this manuscript has undergone a meticulous language revision and proofreading as you have recommended it. We intentionally maintained a descriptive writing style, to facilitate the reproducibility of our experimental methods and results by other researchers in the field. Our aim was to provide a comprehensive account of our findings, prioritizing accuracy, and detail.
